# Influence of operative timing on perioperative outcomes of patients with the seatbelt sign

**DOI:** 10.1016/j.sopen.2023.04.005

**Published:** 2023-04-18

**Authors:** Nolan M. Winicki, Isabella S. Florissi, Alberto Nunez, Jeremy Santiago, Sigrid Burruss, Daniel P. Srikureja

**Affiliations:** a University of California Riverside, School of Medicine, Riverside, CA, United States of America; b Loma Linda University, Department of Surgery, Loma Linda, CA, United States of America; c Laboratory of Cardiovascular Science, National Institute of Health, Baltimore, MD, United States of America; d Johns Hopkins University, School of Medicine, Baltimore, MD, United States of America; e Beacon Medical Group Trauma & Surgical Services, South Bend, IN, United States of America

**Keywords:** Seatbelt sign, Intra-abdominal injury, Laparotomy, Perioperative outcomes

## Abstract

**Background:**

The seatbelt sign (SBS) is a pattern of bruising/contusions on the chest and abdominal wall following motor vehicle collisions. The aim of this analysis is to investigate the influence of time to surgery following identification of the SBS on perioperative outcomes.

**Methods:**

A retrospective review of the Trauma Quality Improvement Program database from 2017 to 2019 was performed. Patients included in this retrospective analysis were involved in motor vehicle collisions, experienced blunt abdominal trauma, presented with skin abrasions/contusions in the SBS distribution, were hemodynamically stable, and underwent laparotomy. Demographics, vital signs, injury severity score, Glasgow coma scale, preoperative CT scans (P-CT), and time from presentation to surgery were recorded. Time from presentation to surgery was subdivided by data quartiles as immediate (<1.3 h), early (1.3–4 h), and delayed (>4 h). The influence of operative timing on postoperative mortality, hospital length of stay (LOS), intensive care unit (ICU) LOS, and ventilator days was assessed in multivariate analyses.

**Results:**

A total of 1523 patients were included; 280 underwent immediate, 610 early, and 633 delayed surgery. Patients undergoing surgery in the early and delayed groups who received P-CT scans had shorter mean times to operation (4.52 h vs 5.24 h, *p* < 0.01). In multivariate analysis, patients who underwent delayed surgery stayed in the hospital 2.5 days longer (*p* < 0.001), spent 2.8 additional days in the ICU (*p* < 0.001), and spent 3.75 additional days on a ventilator (*p* < 0.001) than patients who received early surgery. Within the early and delayed surgical groups, P-CT was associated with lower mortality (OR 0.46 95 % CI 0.24–0.88, *p* < 0.01) in multivariate analysis.

**Conclusions:**

Early surgical intervention was associated with improved patient outcomes by reducing hospital and ICU LOS and ventilator days. Conducting P-CT reduced the time to surgery and mortality. Utilization of P-CT for screening hemodynamically stable patients with the SBS upon admission may expedite identification of the potential need for surgical management of abdominal injury.

## Introduction

Per year in the United States, there are 181 motor vehicle collisions per 100 million vehicle-miles, injuring 79, and killing 1.34 people per 100 million vehicle-miles [1]. Seatbelt use during motor vehicle collisions (MVC) is associated with decreased risk of injury and/or mortality during collisions [2]. Seatbelts can leave a pattern of bruising and burning on the chest and abdominal wall following MVC, and consequently compress internal organs due to abrupt deceleration against the seatbelt. This pattern of bruising is termed the seatbelt sign (SBS) and is strongly associated with visceral organ and intra-abdominal injury [[3], [4], [5]], especially if the SBS is located above the anterior superior iliac spine [6].

The risk of abdominal injury in the setting of high-speed MVCs is high and may be missed at initial presentation [7]. While hemodynamically unstable patients, defined as those with systolic blood pressures <90 and heart rates >100, are stabilized and treated emergently for their injuries, hemodynamically stable patients with abdominal injury may experience delayed care [8]. As such, the presence of the SBS, especially in hemodynamically stable patients, should raise suspicion for underlying organ damage that needs further evaluation through a variety of diagnostic modalities. Computed tomography (CT), is the preferred diagnostic tool, and is extremely sensitive for identifying intra-abdominal and hollow viscus injury in patients with the SBS [9,10]. Focused assessment with sonography in trauma (FAST), confers a lower sensitivity in detecting abdominal injury, and literature shows that negative findings from FAST should be subsequently confirmed with CT [11].

The current literature in the realm of treating patients presenting to emergency services following MVC with an SBS has focused on defining the optimal surgical management in hemodynamically unstable patients [12]. However, criteria regarding the optimal timing for surgical intervention has not been defined. The primary aim of this analysis is to investigate the impact of time to surgery on perioperative outcomes of hemodynamically stable patients with a SBS presenting to the emergency room.

## Methods

### Data/study population

We conducted a retrospective study using the Trauma Quality Improvement Program (TQIP) database, a dataset maintained by the American College of Surgeons that collects data on more than 875 trauma centers across the United States [13]. We identified all patients with MVCs from 2017 to 2019 who fulfilled the following criteria: 1) experienced blunt abdominal trauma, 2) were confirmed to be wearing both a lap and chest seatbelt at the scene of the accident, 3) presented with soft tissue skin contusions/abrasions in the “seatbelt sign” distribution on both the abdomen and anterior thorax, 4) were alive in the emergency room upon arrival, 5) were hemodynamically stable (defined as systolic blood pressure >90 mm Hg and a heart rate under 100 beats/min) [8], and 6) underwent laparotomy using the International Classification of Diseases, Tenth Revision codes [14].

### Data collection & outcomes

Variables collected included demographics, vital signs on admission, injury severity score (ISS) [15], Glasgow coma scale score (GCS) [16], completion of a preoperative CT scan and/or FAST exam, administration of blood products, method of transport to the hospital, hospital characteristics (including trauma verification level, bed size, financial status, and teaching status), time from emergency department admission to surgery, intensive care unit (ICU) length of stay (LOS), hospital LOS, days spent on ventilator, and in-hospital mortality. The data quartiles (Q) were calculated based on the time from admission to surgery: Q1 (1.3) Q2 (2.2) Q3 (4.0). Median and IQR were used due to the apparent non-Gaussian distribution of the data in which utilization of a mean ± standard deviation to divide the cohorts would lead to potentially inaccurate cutoffs that are not truly representative of the data stratification. Based on clinical and sample size considerations we decided to combine Q2 and Q3 to create three groups. The cutoffs used were Q1 (1.3) and Q3 (4.0) in which three groups were generated. These groups were patients who underwent: Immediate (<1.3 h), Early (between 1.3 and 4 h) and Delayed (>4 h) surgery. These cutoffs were also compared to the Loma Linda Institutional guidelines in which a delayed time to operation is set as greater than 4 hours.

### Statistical analysis

All statistical analyses were performed using GraphPad Prism Version 9.2.0 (GraphPad Software, San Diego, California USA). Data are presented as median (interquartile range) for continuous variables, unless otherwise indicated. Categorical variables were compared using Chi-squared test with Yate's continuity correction or Fisher's exact test with doubled one-sided *p* values when appropriate. Continuous variables were compared using the Student's *t*-test or the Mann-Whitney *U* test depending on the normality of data distribution. Multivariate linear and logistic regressions were performed to assess the relation of age, gender, systolic blood pressure, pulse rate, ISS, GCS, method of hospital transport, completion of preoperative CT and/or FAST exam, administration of any blood products, timing to surgery (Immediate, Early, Delayed), hospital profit status, hospital bed size, hospital teaching status, and hospital trauma level on hospital LOS, ICU LOS, days spent on ventilator, and hospital mortality. Data were checked for multicollinearity with the Belsley-Kuh-Welsch technique. Heteroskedasticity and normality of residuals were assessed respectively by the White test and the Shapiro-Wilk test. Patients with missing data were excluded from the analysis. The Newey West correction for heteroskedasticity was applied. A *p*-value < 0.05 was considered statistically significant.

## Results

### Patient demographics

A total of 234,858 patients presented to the hospital alive after a MVC with blunt abdominal trauma during the study period. Patients without complete documentation of a seatbelt sign and full inclusion criteria were excluded (N = 233,335). A total of 1523 patients were included in this analysis; 280 patients underwent immediate, 610 early, and 633 experienced delayed surgery (Table 1). Patients receiving immediate surgery presented with higher ISS, lower GCS, less often utilized preoperative CT scans, utilized more blood products, and displayed a higher mortality than patients who received early and delayed surgery. There were no significant differences between the early and delayed groups in ISS, GCS, and vitals upon presentation.

**Table 1:** Patient characteristics.

	All patients (N = 1523)	Immediate surgery (N = 280)	Early surgery (N = 610)	Delayed surgery (N = 633)	*p* value
Age	35 (22–58)	40 (25–59)	38 (22–59)	32 (21–57)	0.060
Female (%)	891 (58.5 %)	184 (65.7 %)	342 (56.1 %)	365 (57.7 %)	<0.05
Systolic blood pressure (mm Hg)	122 (107–138)	113 (98–130)	123 (107–140)	121 (110–140)	<0.001
Pulse rate	94 (19–111)	94 (80–114)	94 (78–112)	93 (79–112)	0.780
BMI	27.6 (23.6–32.3)	26.9 (24.3–30.7)	27.6 (24.0–32.7)	27.9 (23.1–32.5)	0.510
Injury severity score	17 (10–26)	26 (17–35)	17 (11–27)	14 (9–22)	<0.001
Glasgow coma scale	15 (15–15)	15 (12–15)	15(14–15)	15(15–15)	<0.001
Transport method					0.250
Ground ambulance	1143 (75.0 %)	203 (72.5 %)	461 (75.6 %)	479 (75.6 %)	
Helicopter ambulance	345 (22.6 %)	73 (26.7 %)	138 (22.6 %)	134 (21.1 %)	
Other	34 (2.2 %)	4 (1.4 %)	11 (1.8 %)	19 (3.0 %)	
Preoperative FAST	513 (33.7 %)	99 (35.4 %)	216 (35.4 %)	198 (31.3 %)	0.250
Preoperative CT	1044 (63.8 %)	148 (45.8 %)	437 (67.1 %)	459 (69.4 %)	<0.001
Preoperative FAST + CT	411 (26.9 %)	63 (22.5 %)	186 (30.5 %)	162 (25.6 %)	<0.001
Blood product administration	433 (28.4 %)	163 (58.2 %)	190 (31.2 %)	80 (12.6 %)	<0.001
Time to OR (hours)	2.30 (1.3–3.9)	0.9 (0.7–1.1)	2.4 (1.8–3.0)	6.1 (4.8–8.4)	<0.001
Hospital LOS (days)	7.6 (4.0–12.8)	7.1 (1.0–14.0)	7.0 (3.1–12.7)	8.0 (5.0–13.1)	<0.001
ICU LOS (days)	5.0 (3.0–8.0)	5.0 (3.0–11.0)	5.0 (3.0–9.0)	6.0 (3.0–11.0)	<0.05
Ventilator days	3.0 (2.0–6.0)	5.0 (2.0–11.0)	3.0 (2.0–8.0)	5.0 (2.0–10.0)	<0.001
Mortality	160 (10.5 %)	56 (20.1 %)	73 (12.0 %)	31 (4.9 %)	<0.001
Hospital characteristics					
Trauma level					<0.05
1	732 (48.1 %)	143 (51.1 %)	277 (45.4 %)	312 (49.3 %)	
2	320 (21.0 %)	53 (18.9 %)	148 (24.3 %)	119 (18.8 %)	
3≥	471 (30.9 %)	84 (30.0 %)	185 (30.3 %)	202 (31.9 %)	
Bedsize					0.920
≤200	103 (6.7 %)	17 (6.1 %)	45 (7.3 %)	41 (6.4 %)	
201–400	404 (26.5 %)	71 (25.3 %)	169 (27.7 %)	164 (25.9 %)	
401–600	448 (29.4 %)	85 (30.3 %)	178 (29.1 %)	185 (29.2 %)	
>600	568 (37.3 %)	107 (38.2 %)	218 (35.7 %)	243 (28.3 %)	
Financial status					0.670
For profit	174 (11.4 %)	33 (11.7 %)	74 (12.1 %)	67 (10.5 %)	
Non-profit	1347 (88.4 %)	247 (88.2 %)	535 (87.7 %)	565 (89.3 %)	
Teaching status					<0.05
University	811 (53.3 %)	170 (60.7 %)	306 (50.2 %)	335 (52.9 %)	
Community	501 (13.8 %)	76 (27.1 %)	204 (33.4 %)	221 (34.9 %)	
Non-teaching	211 (13.9 %)	34 (12.1 %)	100 (16.4 %)	77 (12.1 %)	

Data listed as median (Q1-Q3) or number of patients (percentage of sample) as appropriate; Body Mass Index, BMI; computed tomography, CT; operating room, OR; length of stay, LOS; intensive care unit, ICU.

There were no statistically significant differences between each patient group in the method of transport to the hospital, hospital trauma level, hospital bed size, and hospital financial status (Table 1). University affiliated hospitals performed a significantly higher proportion of immediate surgery (Immediate 60.4 %; Early 50.4 %; Delayed 52.8 %, *p* < 0.01) compared to community and non-teaching hospitals.

Blood products were most frequently administered for patients undergoing immediate surgery (58.2 % Immediate; 31.2 % Early; 12.6 % Delayed, *p* < 0.001) (Table 1). Preoperative CT scans were less frequently administered in patients receiving immediate surgery and there was no significant difference in rates of receipt of preoperative CT scans between the early and delayed groups (47.5 %, Immediate; 68.7 % Early; 69.5 % Delayed, *p* = 0.890). There was no statistically significant difference in the utilization of the FAST abdominal exam by surgical timing group.

Median hospital LOS in days differed significantly by surgical timing group (Immediate 7.1 (1.0–14.0); Early 7.0 (3.1–12.7); Delayed 8.0 (5.0–13.1), *p* < 0.001) (Table 1). Median ICU stay in days was longer in the delayed surgery group, as compared to the early surgery group (Early 5.0 (3.0–9.0); Delayed 6.0 (3.0–11.0), *p* < 0.05). The median number of days spent on a ventilator was significantly lower in patients who received early surgery, as compared to those who received immediate and delayed surgery (Immediate 5.0 (2.0–11.0); Early 3.0 (2.0–8.0); Delayed 5.0 (2.0–10.0), *p* < 0.001). Mortality was significantly higher in the immediate surgery group, compared to early and delayed (Immediate 20.1 %; Early 12.0 %; Delayed 4.9 %, *p* < 0.001).

### Influence of imaging modality on time to surgery

Patients were stratified based on whether they received no imaging prior to operation, a preoperative CT, a FAST exam, or a FAST exam and CT. The time from admission to the operating room (OR) was compared for patients in the early and delayed surgical groups, stratified by imaging modality. Patients who underwent CT or a FAST exam and CT prior to surgery were brought to the OR more rapidly than those who had no imaging (mean ± SD hours: None (5.24 ± 5.01), CT (4.52 ± 3.68), FAST and CT (3.64 ± 2.48); *p* < 0.01). There was no statistically significant difference in time to the operating room between patients who received isolated FAST exams (N = 513) and those who received no preoperative imaging (N = 320) (isolated FAST 5.36 ± 5.08 vs no preoperative imaging 5.24 ± 5.01; *p* = 0.991).

### Influence of hospital characteristics on time to surgery

Hospital characteristics of trauma certification level, bed size, financial status, and teaching status were compared to assess if there was an influence on the time from admission to the OR (Table 1). Patients who were treated at a Level 2 trauma center exhibited a shorter delay to operation compared to those treated at Level 3 centers or higher. There was no statistically significant difference in time to operation between Level 1 and Level 2 centers (mean ± SD hours: Level 1 (3.09 ± 2.52), Level 2 (2.70 ± 2.00), Level ≥ 3 (3.48 ± 3.11), *p* < 0.001). There was no significant difference between hospitals of different bed sizes in the time from admission to surgery.

The hospital's financial status was associated with differences in the time to surgery. Patients receiving care at for-profit hospitals received surgery more promptly than those cared for at non-profit hospitals (mean ± SD hours: for-profit (2.71 ± 1.92), non-profit (3.19 ± 2.73), *p* < 0.05). Patients treated at community hospitals exhibited the longest delays to surgery, with time from admission to surgery longer than those experienced in both university and non-teaching hospitals (mean ± SD hours: Community (3.56 ± 3.31), University (3.01 ± 2.59), non-Teaching (2.69 ± 1.73), *p* < 0.001).

### Multivariate analysis

A multivariate analysis, adjusting for demographics, transportation method, admitting hospital characteristics, imaging modalities, injury severity, and clinical status upon admission was conducted to predict hospital LOS, ICU LOS, total ventilator days, and in-hospital mortality (Table 2, Table 3).

**Table 2:** Multivariate predictive factors for hospital length of stay, ICU length of stay and total ventilator days.

Variable	Hospital length of stay		ICU length of stay		Ventilator days	
	Coefficient	*p*	Coefficient	*p*	Coefficient	*p*
Age	0.01 [−0.03;0.04]	0.710	−0.03 [−0.07;0.01]	0.180	−0.02 [−0.06;0.03]	0.412
Sex (ref. female)						
Male	**1.81 [0.47;3.34]**	**<0.01**	0.22 [−1.09;1.53]	0.740	−0.14 [−1.5;1.22]	0.837
Systolic blood pressure	**−0.04 [−0.07;-0.01]**	**<0.001**	−0.02 [−0.06;0.01]	0.122	−0.02 [−0.05;0.02]	0.321
Pulse rate	0.02 [−0.01;0.05]	0.170	−0.01 [−0.04;0.03]	0.772	−0.01 [−0.04;0.04]	0.878
Body Mass Index	**0.14 [0.05;0.23]**	**<0.01**	**0.21 [0.08;0.31]**	**<0.001**	**0.16 [0.03;0.27]**	**<0.05**
Injury severity score	**0.22 [0.15;0.30]**	**<0.001**	**0.20 [0.07;0.31]**	**<0.001**	**0.12 [0.01;0.21]**	**<0.05**
Glasgow coma scale	0.11 [−0.77;0.99]	0.800	0.29 [−0.45;1.02]	0.445	−0.07 [−0.39;0.25]	0.681
Transport method (ref. ground ambulance)						
Helicopter ambulance	0.89 [−0.85;2.63]	0.310	−0.06 [−1.59;1.47]	0.939	0.78 [−0.87;2.42]	0.354
Other	3.38 [−2.65;9.42]	0.270				
Hospital trauma level (ref. 1)						
2	−1.37 [−3.66;0.91]	0.240	−1.49 [−3.44;0.46]	0.133	0.34 [−1.82;2.5]	0.756
≥3	−0.68 [−2.46;1.10]	0.450	−0.37 [−1.99;1.26]	0.657	0.21 [−1.58;2.0]	0.815
Hospital financial status (ref. non-profit)						
For profit	−0.69 [−2.98;1.60]	0.550	−1.2 [−3.68;1.28]	0.344	**−3.33 [−5.04;-1.62]**	**<0.001**
Hospital teaching status (ref. non-teaching)						
Community	1.25 [−1.13;3.62]	0.300	−1.46 [−3.78;0.86]	0.218	0.24 [−2.07;2.55]	0.838
University	1.46 [−1.07;3.99]	0.260	−0.21 [−2.78;2.38]	0.879	1.73 [−1.25;4.71]	0.255
Hospital bedsize (ref. ≤200)						
201–400	0.03 [−3.02;3.09]	0.980	0.45 [−2.15;3.05]	0.732	2.67 [−0.12;5.46]	0.061
401–600	0.40 [−2.68;3.47]	0.810	0.09 [−3.07;3.25]	0.956	1.93 [−1.38;5.24]	0.253
>600	−1.15 [−4.23;1.93]	0.470	−1.81 [−4.57;0.95]	0.197	0.02 [−2.67;2.71]	0.989
Blood products	0.73 [−1.16;2.62]	0.450	**2.76 [0.26;5.26]**	**<0.05**	1.82 [−0.36;4.01]	0.102
Preoperative FAST	0.73 [−0.84;2.30]	0.360	0.42 [−0.89;1.73]	0.530	0.29 [−1.38;1.98]	0.730
Preoperative CT	**2.23 [0.69;3.70]**	**<0.01**	−0.48 [−2.14;1.04]	0.538	−0.26 [−2.09;1.57]	0.780
Preoperative FAST + CT	−0.16 [−3.25;2.92]	0.920	−0.49 [−3.46;2.49]	0.750	−1.05 [−4.33;2.22]	0.530
Surgery timing (ref. early)						
Immediate	−0.39 [−2.53;1.75]	0.720	−0.30 [−2.48;1.89]	0.790	−1.34 [−3.06;0.39]	0.260
Late	**2.58 [1.01;4.20]**	**<0.001**	**2.63 [0.89;4.40]**	**<0.001**	**3.29 [1.44;6.06]**	**<0.001**

Bold data indicate statistical significance.

**Table 3:** Multivariate predictive factors for mortality.

Variable	Mortality	
	Odds ratio	*p*
Age	1.01 [0.99;1.02]	0.148
Sex (ref. female)		
Male	1.06 [0.60;1.85]	0.850
Systolic blood pressure	0.99 [0.99;1.01]	0.419
Pulse rate	0.99 [0.98;1.01]	0.298
Body Mass Index	**1.05 [1.02;1.09]**	**<0.001**
Injury severity score	**1.07 [1.05;1.11]**	**<0.0001**
Glasgow coma scale	1.16 [0.85;1.59]	0.560
Transport method (ref. ground ambulance)		
Helicopter ambulance	0.73 [0.38;1.39]	0.336
Hospital trauma level (ref. 1)		
2	0.61 [0.23;1.64]	0.326
≥3	0.93 [0.45;1.92]	0.845
Hospital financial status (ref. non-profit)		
For profit	1.96 [0.89;4.34]	0.097
Hospital teaching status (ref. non-teaching)		
Community	0.64 [0.26;1.57]	0.327
University	0.45 [0.17;1.23]	0.118
Hospital bedsize (ref. ≤200)		
201–400	0.55 [0.20;1.53]	0.253
401–600	0.38 [0.13;1.13]	0.082
>600	0.76 [0.27;2.15]	0.609
Blood products	**4.37 [2.29;8.35]**	**<0.0001**
Preoperative FAST	1.57 [0.82;3.04]	0.180
Preoperative CT	**0.43 [0.23;0.78]**	**<0.001**
Preoperative FAST + CT	0.64 [0.20;2.03]	0.450
Surgery timing (ref. early)		
Immediate	0.64 [0.32;1.27]	0.270
Late	0.91 [0.48;1.75]	0.610

Bold data indicate statistical significance.

### Hospital length of stay

Male gender (coefficient of effect [95 % confidence interval] 1.81 [0.47;3.34], *p* < 0.01), higher BMI (0.13 *per point increase* [0.04;0.23], *p* < 0.01), and higher ISS (0.22 *per point increase* [0.15;0.30], *p* < 0.0001) were associated with increased hospital LOS (Table 2). Receipt of preoperative CT scan was associated with a longer hospital LOS (2.23 [0.69;3.70], *p* < 0.001). Patients who underwent delayed surgery experienced a longer hospital LOS than those who underwent early surgery (2.58 [1.01;4.20], *p* < 0.001).

### ICU length of stay

Higher BMI (0.21 *per point increase* [0.08;0.31], *p* < 0.001), higher ISS (0.20 *per point increase* [0.07;0.31], *p* < 0.001), and transfusion of blood products (2.76 [0.26;5.26], *p* < 0.05) were associated with an increased ICU LOS (Table 2). Patients who underwent delayed surgery experienced longer ICU stays than those who underwent early surgery (2.63 [0.89;4.40], *p* < 0.001).

### Days spent on ventilator

Higher BMI (0.16 *per point increase* [0.03;0.27], *p* < 0.05) and higher ISS (0.12 *per point increase* [0.006;0.21], *p* < 0.05) were associated with longer total days spent on a ventilator (Table 2). Receipt of care at a non-profit hospital, compared to a for-profit, was associated with shorter time spent on a ventilator (−3.33 [−5.04;−1.62], *p* < 0.0001). Delayed surgery was associated with more ventilator days compared to patients who underwent early surgery (3.29 [1.44;6.06], *p* < 0.001).

### Mortality

A higher BMI (OR 1.05 *per point increase* [95 % CI] [1.02;1.09], *p* < 0.001) and higher ISS (OR 1.07 *per point increase* [1.05;1.11], *p* < 0.0001) were associated with an increased risk of mortality (Table 3). Administration of blood products was associated with a higher risk of mortality (OR 4.37 [2.29;8.35], *p* < 0.0001). Receipt of preoperative CT scan was associated with a lower risk of mortality (OR 0.43 [0.23;0.78], *p* < 0.001).

To account for the higher incidence of mortality in the immediate surgical group, the influence of preoperative CT on mortality was assessed within only the early and delayed groups. Receipt of preoperative CT was associated with a decreased incidence of mortality in both the early and delayed groups (OR 0.46 [0.24;0.88], *p* < 0.01). There was no statistically significant difference in mortality between surgical timing groups (Table 3).

## Discussion

Our study explored timing of surgical intervention for hemodynamically stable patients who presented to emergency services following a MVC with the SBS. We found that early intervention (within 1.3–4 hours of admission) was associated with a decreased hospital LOS, ICU LOS, and total days spent on a ventilator. Additionally, we found that screening patients with either an abdominal CT or a FAST scan followed by a confirmatory CT reduced the time from admission to the operating room and thus improved patient outcomes. No benefit was seen in time to operating room for FAST only in hemodynamically stable patients with a seatbelt sign.

In this study, we utilized the TQIP database maintained by the American College of Surgeons which includes patient data from more than 875 participating trauma centers across the US. This study, to our knowledge, contains the largest known cohort of patients who suffered a MVC, presented to emergency services with the SBS, and required laparotomy during their hospital admission. We aimed to determine the optimal timing of surgical intervention to guide patient care and improve outcomes.

Hemodynamically unstable patients require urgent interventions; thus, we chose to focus our analysis on patients who are hemodynamically stable at initial presentation. Hemodynamically stable patients with SBS experience varied methods of screening, observation, and indications for non-emergent operation, and the protocol of care received is largely dependent on the institution where the care is offered [1,2]. Literature on management of patients with SBS after MVC who are hemodynamically stable is limited. A previous study, exploring a smaller cohort of patients experiencing the SBS following a MVC, investigated the impact of operative timing on the care of abdominal injuries with similar median times of 1.05, 2.7 and 19.5 hours for their immediate, early and delayed groups, respectively [3]. However, this analysis included hemodynamically unstable patients in the immediate surgical group and represented management from a sole institution [3]. In our analysis, we found that patients who underwent immediate surgery (<1.3 hours from presentation) presented with higher ISS, less often utilized preoperative CT scans, received more blood products perioperatively, and displayed a higher mortality than those that received early and delayed surgery. However, GCS, ISS and vitals upon presentation did not differ significantly between patients who received early and delayed surgery. Hospital characteristics such as being for-profit and a University or non-teaching hospital compared to community hospital was associated with decreased time from admission to surgery, however these differences did not impact patient outcomes when examined in the multivariate analysis.

Diagnostic options for patients presenting with blunt abdominal trauma include a FAST ultrasound exam, CT scans of the abdomen, and diagnostic peritoneal lavage [4]. Recent studies of patients with the SBS have shown strong utility of CT scans of the abdomen in the diagnosis of intra-abdominal injury and in identifying the need for urgent intervention [1,5]. CT scans have become the primary diagnostic tool for blunt abdominal trauma in hemodynamically stable patients, with an accuracy of over 95 % and negative predictive value of almost 100 % [4]. In our analysis, we found that receipt of preoperative CT was associated with a decreased time to operation, and that receipt of a FAST exam confirmed by CT expedited the time to surgery, as compared to patients who received no diagnostic imaging. The administration of a sole FAST exam without follow-up CT did not show any benefit in time to surgery, as compared to no imaging. This is likely a consequence of the low sensitivity of FAST for detecting blunt intra-abdominal injury and a FAST only for patients with a seatbelt sign is not indicated. In fact, previous studies have found that many intra-abdominal injuries were missed in patients who received only FAST imaging [6,7].

Previous studies have shown improvement in the sensitivity of the FAST exam in identifying free intra-peritoneal fluid, especially when followed by a repeated ultrasound exam, but these results are less accurate than abdominal CT scans [8,9]. For all patients presenting with significant seatbelt abrasions or contusions, initial CT has been shown to be 100 % sensitive for detecting abdominal injury [5]. With this support from previous literature, and with the findings in our study, it is evident that screening hemodynamically stable patients with an SBS following a MVC with an initial CT could decrease delays to operation, shown in our study to improve patient outcomes. Moreover, negative CTs can potentially increase safe discharges in patients with the SBS who do not require surgery due to lack of intra-abdominal injury [1,10,11].

We found that delayed intervention influences the morbidity of patients suffering blunt abdominal trauma following MVC with a seatbelt sign. In concordance with our results, previous studies have not reported a significant difference in mortality for patients with abdominal trauma and a positive SBS following MVC who receive early or delayed surgical intervention [[12], [13], [14], [15], [16]]. However, we observed significant differences in hospital LOS, ICU LOS, and total ventilator days in the delayed surgery group. Other reports of surgical intervention for blunt bowel injury have shown similar findings, with delayed surgery (treatment after 8 hours) corresponding to increased hospital LOS [15,16]. Our cutoff for delayed surgery was intervention after 4 hours, suggesting that there may be a smaller window of timely care than previously demonstrated. Outside of surgical timing, consistent risk factors for morbidity and mortality in our study were higher patient BMIs and ISS. Interestingly, morbid obesity has shown conflicting results in patients with blunt abdominal trauma. Morbid obesity has been shown to be a protective factor for injury to hollow viscus organs, but also has shown to increase the risk of morbidity and mortality following trauma [17,18]. As expected, an increased ISS was associated with longer hospital LOS and increased mortality, as previously validated [19].

There are several limitations to our study. First, the TQIP database does not contain a specific variable that validates whether patients presented to emergency care with a seatbelt sign. Instead, we used the following conjunction of variables as a proxy: confirmation that both a lap and chest seatbelt were worn at the accident scene, as well as noted skin contusions and ecchymosis on both the abdomen and anterior thorax. While we believe these variables accurately represent the SBS, we acknowledge that patients with a less classic presentation of the SBS may have been excluded from our analysis. As a database study, there may be uncaptured variables that may confound our findings and the imaging findings are not known. Moreover, as this is a retrospective study, we are unable to establish causality in our results. Lastly, the time frame of the study is limited. Nonetheless, this is the largest cohort to date, to our knowledge, to study victims of MVC with SBS at presentation to emergency services and can significantly impact the quality of care delivered to patients suffering abdominal trauma following MV collisions.

Early surgical intervention before 4 hours after a MVC in patients with a SBS who are hemodynamically stable was associated with improved patient outcomes by reducing hospital LOS, ICU LOS, and total days spent on a ventilator. Additionally, conducting a preoperative abdominal CT reduced the time from admission to surgery, and showed reduced mortality in multivariate analysis whereas only a FAST exam provided no benefit to the patient and time to surgery. We recommend that hemodynamically stable patients with a SBS should be screened with abdominal CTs at the time of presentation to the ED to identify the potential need for and expedite the surgical management of abdominal injury.

## Funding sources

This research did not receive any specific grant from funding agencies in the public, commercial, or not-for-profit sectors.

## Ethical approval statement

This study was exempt from institutional review board approval at Loma Linda University as the ACS-TQIP is a publicly available, deidentified database.

## CRediT authorship contribution statement

**Nolan M. Winicki:** Conceptualization, Methodology, Investigation, Formal analysis, Writing – original draft. **Isabella S. Florissi:** Data curation, Writing – review & editing. **Alberto Nunez:** Data curation, Writing – review & editing. **Jeremy Santiago:** Data curation, Writing – review & editing. **Sigrid Burruss:** Conceptualization, Methodology, Data curation, Writing – review & editing, Supervision. **Daniel P. Srikureja:** Conceptualization, Methodology, Data curation, Writing – review & editing, Supervision.

## Competing interest

The authors have no significant competing interests.

## Data Availability

The data that support the findings are available from the corresponding author upon reasonable request.
